# Experiences of participants with undiagnosed diseases and hereditary cancers during the initial phase of the Hong Kong genome project: a mixed-methods study

**DOI:** 10.1186/s40246-025-00746-5

**Published:** 2025-04-05

**Authors:** Annie TW Chu, Samuel YC Sze, Desiree MS Tse, Cheryl WY Lai, Carmen S Ng, Coco WS Yu, Pui-hong Chung, Fei-chau Pang, Brian HY Chung, Su-vui Lo, Jianchao Quan

**Affiliations:** 1 Hong Kong Genome Institute, Hong Kong SAR, China; 2 School of Public Health, LKS Faculty of Medicine, The University of Hong Kong, Hong Kong SAR, China; 3 Health Bureau, Hong Kong SAR, China; 4 Department of Paediatrics and Adolescent Medicine, LKS Faculty of Medicine, The University of Hong Kong, Hong Kong SAR, China; 5 HKU Business School, The University of Hong Kong SAR, Hong Kong SAR, China

**Keywords:** Genome, Program evaluation, Rare diseases, Hereditary cancer, Patient experience, Satisfaction, China

## Abstract

**Background:**

The Hong Kong Genome Project (HKGP) is the first population-wide whole genome sequencing (WGS) programme in Hong Kong and aimed to integrate genomic medicine into the healthcare system. Implementing genetic counselling is essential to help participants understand the genetic basis of diseases and guide informed decision making. We assessed participant experiences during the initial HKGP pilot phase that enrolled patients with undiagnosed diseases and hereditary cancers.

**Methods:**

Participants were recruited from three partnering centres at public hospitals during June-September 2023. Participant surveys covered four domains: (1) overall satisfaction, (2) informed consent process, (3) genetic counselling, and (4) attitude towards HKGP. Associations with demographic and socioeconomic characteristics were assessed with multivariable logistic regression. Qualitative feedback was collected in focus group interviews.

**Results:**

Among 422 eligible participants, 341 completed the survey (80.8% response) and five focus group interviews were held (21 participants). We found 89.8% [95% CI: 86.1–92.7] were satisfied with their HKGP experience. Almost all felt that HKGP participation could benefit others (86.8% [95% CI: 82.7–90.0]) and advance genomic research in Hong Kong (88.9% [95% CI: 85.0-91.9]). The survey item with the lowest agreement among respondents was feeling that HKGP participation could improve their/child’s medical treatment (73.5% [95% CI: 68.5–78.0]). Those with secondary and tertiary education were less likely to agree genetic counselling was helpful (Odds Ratio [OR]: 0.02 [95% CI: 0.001–0.41]; 0.02 [0.001–0.51]), or the appropriate length of time (OR: 0.12 [95% CI: 0.014–0.81]; 0.11 [0.01–0.91]). Focus group participants cited helping scientific advances and shortening the diagnostic odyssey of future patients as key reasons for participation. Participants hoped for a shorter reporting time of WGS results, additional medical follow-up, and allowing referral of relatives.

**Conclusions:**

Participants were overall highly satisfied with the HKGP and genetic counselling experience. Satisfaction levels were comparable to overseas genomic programmes and locally provided healthcare services. Participants’ major concerns on WGS reporting time could be addressed by strengthening the informed consent process to ensure their expectations align with project implementation. Emphasizing the long-term value of genomic research and its potential for personalized treatments may increase participant engagement.

**Supplementary Information:**

The online version contains supplementary material available at 10.1186/s40246-025-00746-5.

## Background

Genomic medicine has emerged as a transformative approach to personalized healthcare and precision medicine, revolutionizing researchers’ and clinicians’ understanding of diseases, genetic risks, and treatment strategies [1–3]. To harness the potential of genomic medicine, numerous large-scale initiatives have been launched worldwide [4], such as the UK 100,000 Genomes Project initiated by Genomics England in 2013 that enabled whole genome sequencing (WGS) for rare inherited diseases [5]. Using WGS can improve diagnoses that would not have been reported by conventional exome testing [6] and discover novel variants [7]. Understanding participants’ experience and concerns would be useful in informing project implementation. Interpersonal and institutional trust in the healthcare provider (NHS) and investment in improving care for the future were the factors that most influenced their decision to enrol in the project [8]. Participants had concerns on potential psychological impact of results, especially among male, Asians, and more religious respondents [9]. The Hong Kong Genome Project (HKGP) was launched in 2019 with HK$1.2 billion (US $150 million) funding from the Health Bureau, Government of Hong Kong SAR. The project plans to recruit 20,000 cases and sequence 40,000–50,000 genomes over a six-year period in two phases, the pilot and main phase [10–11]. For the initial pilot phase, the target participants were 2,000 cases with undiagnosed diseases and hereditary cancers (genetic predisposition to cancer), and their family members [11]. Participants enrolled in the HKGP receive access to WGS that could facilitate more personalized treatment, in addition to their usual clinical care. In future, the HKGP seeks to establish a comprehensive genomic database and biobank of the local Chinese population [12–13] to facilitate innovative scientific research and the integration of genomic medicine into routine care and the local healthcare system.

The HKGP recruitment process partnered with the existing public healthcare systems to ensure equitable access to genomics. For the pilot phase, three HKGP Partnering Centres were established at the university-affiliated public teaching hospitals to recruit participants, provide genetic counselling, collect samples, handle enquiries, deliver genome sequencing results, and liaise with the hospital clinical teams. Clinicians assessed patients visiting the partnering centres for HKGP enrolment eligibility. All eligible participants were seen by genetic counsellors and received explanations on potential genetic predispositions leading to existing conditions, potential treatment options, family pedigree, reproductive risks and options, prior to obtaining informed consent and sample collection. Further details of the HKGP is provided in Appendix 1 and has been published elsewhere [11].

Recent studies highlight the importance of participant-centred approaches in establishing large-scale genomic projects. A crucial area is the informed consent process that ensure participants are well-informed about the purpose, benefits, and potential risks associated with genomic testing [14], and thus allow incorporation of their preferences in the decision-making process [15]. Previous studies in genetic counselling and clinical genetics settings have highlighted the complexity of the informed consent process and the need for clear communication between participants and genetic counsellors [15–17]. For example, participants in the UK 100,000 Genomes Project had difficulties understanding the complex terminology during the informed consent process, leading to a lack of recall regarding previously made decisions [17]. Counsellors play a critical role in explaining the study process and WGS procedure in a manner that is easily comprehensible, devoid of technical jargons and ensures sufficient understanding. Participants and their family members are made aware of potential implications of their decision for the participants. Additionally, addressing privacy and data security concerns is paramount to maintaining participant trust and ensuring responsible use and protection of genomic data [19]. Striking a balance between data sharing for scientific advancement and safeguarding participant privacy is a critical and dynamic consideration in the implementation of genomic research projects.

Genomic research has been more limited in the Asia relative to other populations, with only 5% of genome-wide association studies (GWAS) conducted on people of East Asian ancestry [20]. The paucity of research examining participant experiences in genome projects and the lack of familiarity with genomics among the general population makes the applicability and acceptability of genomic medicine challenging in any large-scale rollouts. Hence, evaluating participant satisfaction and experiences during the initial phase of a project is crucial for informing policymakers and managers, enabling them to better plan implementation of the main phase and facilitate the integration of genomic medicine into the local healthcare system. In our study on the initial pilot phase of HKGP that enrolled patients with undiagnosed diseases and hereditary cancers, we assessed participant satisfaction across multiple domains, investigated associations with socioeconomic factors, and explored ideas, concerns, and areas for improvement to enhance the future implementation of genomic medicine initiatives.

## Methods

### Study design and participants

We used a mixed-methods study design to assess both quantitative and qualitative aspects of participant experiences during the initial phase of HKGP. The HKGP participant workflow from patient registration to blood sample collection at the three partnering centres is shown in Appendix Fig. 1. For quantitative data collection, we conducted a survey of HKGP participants recruited at enrolment from 13 June to 27 September 2023. The target sample size was 287 based on a finite population of 2,500 HKGP initial phase participants with undiagnosed disease or hereditary cancer, and an assumed 70 ± 5% of satisfaction rate. Participants had potential diagnoses ranging from monogenic cause to more complex aetiology to test the broad utility of WGS, whereas patients with recognisable conditions, or syndromes where genetic testing offers no additional benefit were excluded from analysis [10]. Eligible HKGP participants were referred by genetic counsellors from the three partnering centres to our research team for study enrolment. For participants under the age of 18, consent and responses were collected from parental guardians or caretakers. Survey respondents were subsequently invited to participate in semi-structured focus group interviews provide additional thoughts. Participants received a gift voucher for completing the quantitative survey (~ USD 6) and focus group (~ USD 13).

**Fig. 1:**
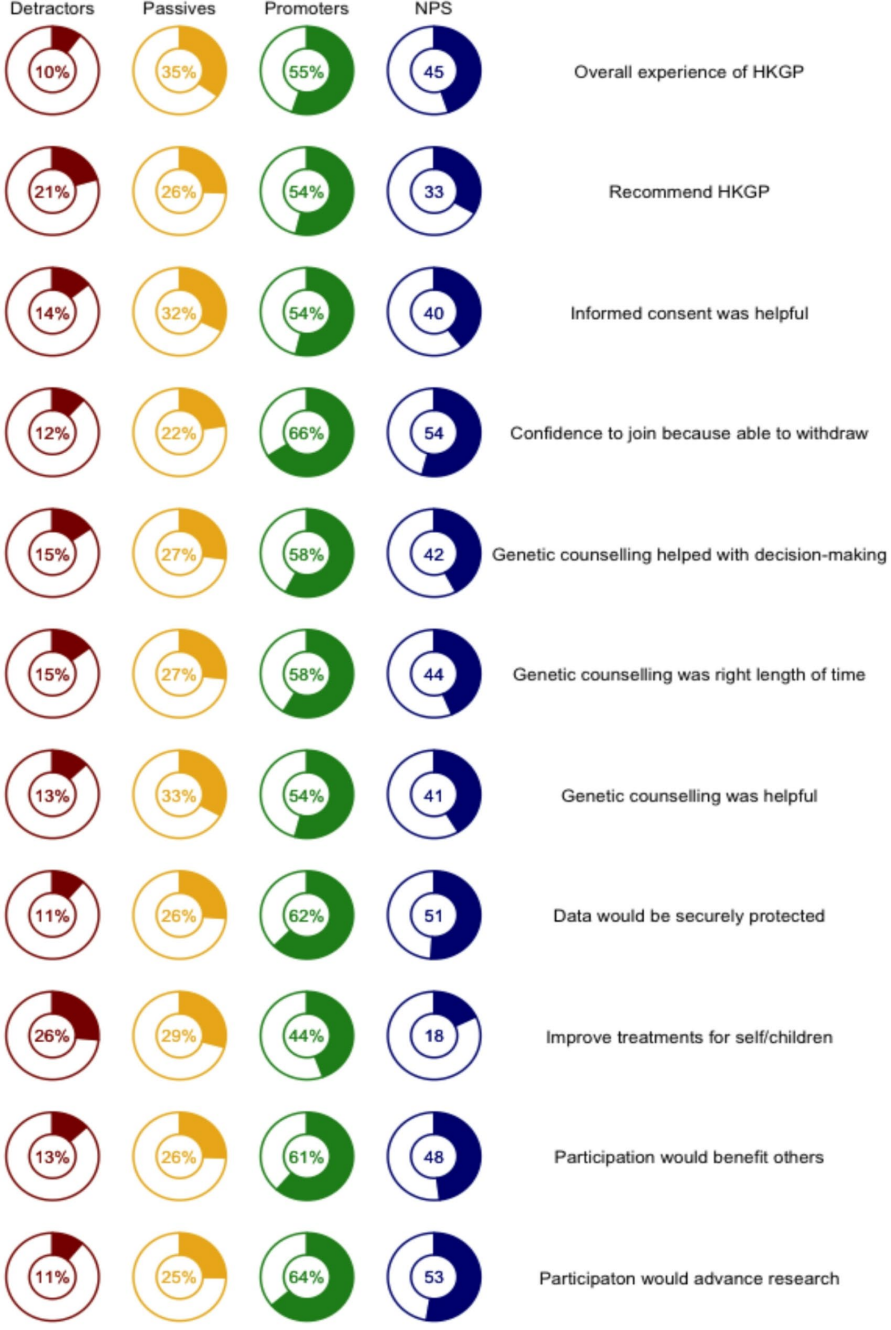
Net Promotor Scores for Patient Satisfaction measures [*n* = 325]

### Design of survey instruments

The design of survey instruments followed our literature review of participant satisfaction surveys for genomic studies that focused on the informed consent and genetic counselling process [15–17]. The initial survey assessed overall satisfaction, patient journey, consent, pre-test genetic counselling, additional (genetic) findings, decision regret, and knowledge/attitude towards WGS and HKGP. Overall satisfaction was based on a previous satisfaction survey for parents of children with rare diseases in the 100,000 Genomes Project [21]. Questions on patient journey were based on the Patient Satisfaction Scale developed by Zellerino et al. [16] that reliably assesses the quality of care and interpersonal relations in the clinical genetics setting. Questions on pre-genetic counselling was derived from the Genetic Counselling Satisfaction Scale developed by DeMarco et al. [18]. that addresses the communication and understanding between participants and genetic counsellors. Section of participants’ decisions on receiving additional findings in their WGS reporting were derived from Ballard et al. [17]. The Decision Regret Scale and Attitudes for patients with rare diseases in the 100,000 Genomes Project [21] was included in our survey. Participant experiences were measured on a five-point Likert Scale. Sociodemographic data on respondents (age, education level, housing, marital status, household income level) were also collected.

The initial survey was reviewed for content validity and feasibility by healthcare professionals (including genetic counsellors, clinical geneticists, paediatricians, HKGP operations team, and the ethics review boards of the three partnering centres). Following feedback, we added questions on the diagnostic odyssey and assessed the value of information packages offered [22]. The decision regret and additional findings sections were omitted as WGS results reporting was unavailable at this pilot stage of HKGP experience. Knowledge of WGS and HKGP was modified to participants’ attitude towards WGS and HKGP similar to previous study on the 100,000 Genomes Project [21]. The final survey was shortened to a ten-minute duration to minimise the burden on participant and improve response rates. Survey questions were translated to traditional Chinese and piloted on HKGP participants (*n* = 28) at a single partnering centre. Wordings on diagnostic odyssey were modified as some participants found it difficult to recall their first clinical consultation that was frequently long ago.

### Quantitative survey

The final survey covered four domains: overall satisfaction with HKGP, informed consent process, genetic counselling, and attitudes towards HKGP (survey form in *Appendix 2*). To minimize recall bias, we aimed to conduct the survey promptly within 30 days after HKGP enrolment. The majority of responses were collected by phone by research staff (92.9%) with 7.1% self-completing the survey online. All data were inputted into Qualtrics XM (Qualtrics, Provo, UT). Participants who did not wish to complete the survey over the phone could self-complete online. Participants received a minimum of five phone calls on five separate days before considered a non-respondent.

### Statistical analysis

Responses on a five-point Likert scale were converted into proportions for ease of understanding and comparison with confidence intervals for satisfaction levels were calculated assuming a binomial distribution. Multivariable logistic regression were performed to assess associations between socioeconomic characteristics and HKGP satisfaction indicators. Responses on five-point Likert scale were converted into binary outcomes of ‘satisfied’ (satisfied and somewhat satisfied) and ‘neutral or dissatisfied’ (dissatisfied, somewhat dissatisfied, and neutral) for the regression models. A similar approach was applied for levels of agreement (agree and somewhat agree vs. disagree, somewhat disagree, and neutral). Model covariates included age, gender, marital status, education, and household income. All tests were two-tailed and a p-value of ≤ 0.05 was considered statistically significant. Analyses were performed using R version 4.3.

We calculated the Net Promoter Score (NPS), developed by Bain & Company, to assess the potential for wider rollout of HKGP and WGS. The NPS measures the overall favourability of participants on the service provided to assess projected service growth (scores > 20 = favourable, > 50 = excellent) [23–24]. We categorized participants responses in five-point Likert scale (promoters = “agree” or “satisfied”, passives = “somewhat satisfied” or “somewhat agree”, detractors = all other responses). The scores were computed by subtracting the percentage of detractors from the percentage of promoters. Calculation of NPS assumed that participants satisfied with the experience would be inclined to advocate to others not yet covered under the service provision scope, while dissatisfied participants might actively discourage others’ from engaging in WGS services.

### Focus groups

Consolidated criteria for reporting qualitative research (COREQ) guidelines were used to structure the research design, analysis, and reporting of findings (Appendix 4) [25]. We used phenomenology as the methodological framework to allow researchers to explore the subjective experiences and opinion of participants [26] and make sense of other’s experiences through interpretative activities [27]. A semi-structured focus group guide (Appendix 3) was designed to explore participants’ perceptions and attitudes on the HKGP. It was developed from preliminary survey findings, previous literature, and experiences from the study team to serve as a reference for probing participant response from the participants, but was not piloted. We aimed to capture contextual factors, reasons leading to the survey responses, and other comments regarding HKGP they wished to express. Survey respondents were randomly stratified and sampled by their gender (male or female) and type (patient or parent/caretaker of patient), and were invited to participate in the focus group. Some participants refused to participate, usually due to being unavailable during the proposed timeslots. Candidates who were willing to participate were distributed across different sessions to ensure diversity of backgrounds in each group. This approach aimed to facilitate discussion and minimize conformity in discussions by allows participants to hear and explore differing experiences. Previous research found that almost all themes or key content were discoverable by conducting three to six focus groups [28], and hence we aimed for five groups to achieve thematic saturation. The group size was limited to six participants to allow opportunities to share. All focus groups were conducted and recorded online from 4 to 15 September 2023 using Zoom to enhance participants accessibility. Sessions lasted around 90 min and were conducted in Cantonese. A researcher who received training and had previous experience conducted the focus group interviews with field notes recorded by two other researchers. Some participants were accompanied by family members, whose responses were not coded and analysed by the researchers.

### Qualitative analysis

A thematic analysis approach was adopted to analyse the qualitative data from the focus groups in five steps: (1) collection and organization of data; (2) obtaining general understanding of information; (3) coding; (4) categorization into themes; and (5) interpretation of results [29]. The focus group sessions were video-recorded and transcribed verbatim with personal identifiers removed. The research team compared all transcripts and recordings to confirm accuracy of the information captured. The video-recordings and transcripts were not provided to participants for reference, feedback or corrections to minimize their burden. Two researchers labelled fundamental patterns on themes and contexts, coded and analysed independently the interview transcripts An open coding strategy was used with no pre-defined themes or codes in a bottom-up approach. Researchers could create new codes upon receiving novel or unexpected responses, with conflicts or alignments in coding resolved after discussion. Concepts repetitively expressed by participants were extracted and presented as illustrative quotes to inform key findings.

## Results

### Quantitative survey

A total of 341 participants from 422 partnering centres referrals completed the survey (response rate of 80.8%). We excluded 15 respondents who completed the survey 30 days after their genetic counselling session, and one respondent with a missing date of enrolment. Thus, 325 completed surveys were included for analysis with 219 (67.4%) completed within one week after enrolment. A flowchart outlining eligible survey participants is provided in Appendix Fig. 2.

Baseline characteristics of survey respondents and focus group participants are listed in Table 1. The HKGP survey participants were evenly spread across age groups with a median age of 47 years old (age range: 1–84). Participants were evenly distributed across gender (50.8% female), and socioeconomic indicators such as income and housing type, though almost all (98.5%) were of Chinese ethnicity. Time elapsed from the first visit or consultation with the healthcare provider varied widely. Most participants (68.0%) first consulted for their condition more than one year ago, 35.1% more than five years ago, and 22.2% over ten years ago.

**Table 1:** Demographic characteristics of survey and interview participants

Characteristics	Survey participants, [*n* = 325] (%)	Interview participants [*n* = 21] (%)
Age group		
≤19	50 (15.4)	4 (19.0)
20–39	81 (24.9)	7 (33.3)
40–59	105 (32.3)	2 (9.5)
≥60	89 (27.4)	8 (38.1)
Gender		
Male	159 (48.9)	7 (33.3)
Female	165 (50.8)	14 (66.7)
Other	1 (0.3)	0 (0)
Ethnicity		
Chinese	320 (98.5)	21 (100)
Other	5 (1.5)	0 (0)
Marital Status		
Married	196 (60.3)	13 (61.9)
Other Marital Status	129 (39.7)	8 (38.1)
Household Income Level (in HKD)		
≤$19,999	35 (10.8)	4 (19.0)
$20,000–$29,999	57 (17.5)	1 (4.8)
$30,000–$39,999	38 (11.7)	3 (14.3)
$40,000–$59,999	49 (15.1)	5 (23.5)
≥$60,000	63 (19.4)	4 (19.0)
(Refused to answer)	83 (25.5)	4 (19.0)
Type of Housing		
Public Housing	108 (33.2)	8 (38.1)
Private Permanent Housing	177 (54.5)	11 (52.4)
Other Types of Housing	29 (8.9)	0 (0)
(Refused to answer)	11 (3.4)	2 (9.5)
Educational level		
Primary or below	58 (17.8)	4 (19.0)
Secondary	135 (41.5)	11 (52.4)
Tertiary or above	132 (40.6)	6 (28.6)
Time from first consultation with healthcare provider for a suspected genetic disorder		
Within a year	104 (32.0)	7 (33.3)
More than a year ago	70 (21.5)	3 (14.3)
More than three years ago	37 (11.4)	4 (19.0)
More than five years ago	42 (12.9)	2 (9.5)
More than ten years ago	72 (22.2)	5 (23.5)

Participant satisfaction or agreement are shown in Table 2. Nine out of eleven indicators in the four domains achieved satisfaction rate over 80%. Most participants were satisfied with their overall experience of the HKGP (89.8%, 95% CI [confidence interval]: 86.1–92.7) and agreed that the genetic counselling session helped them to identify what they needed to know to make decisions affecting themselves (84.6% [95% CI: 80.3–88.1]). Most participants (79.4% [95% CI: 74.7–83.4]) would recommend fellow patients or those with similar needs to participate in the HKGP. The lowest level of agreement was belief that taking part in the HKGP could improve their personal/child’s medical treatment (73.5% [95% CI: 68.5–78.0]); though more felt that taking part in HKGP could benefit others (86.8% [95% CI: 82.7–90.0]) and advance genomic research in Hong Kong (88.9% [95% CI: 85.0-91.9]).

**Table 2:** Satisfaction of HKGP participants [*n* = 325]

Indicator	Disagree/ Dissatisfied (%)	Somewhat disagree/ dissatisfied (%)	Neutral (%)	Somewhat agree/ satisfied (%)	Agree/ Satisfied (%)	Agree/ Satisfied to any extent % [95% CI]
How Would You Rate Your Overall Experience of the Hong Kong Genome Project (HKGP) Thus Far?	0 (0)	2 (0.6)	31 (9.5)	113 (34.8)	179 (55.1)	89.8 [86.1, 92.7]
You Would Recommend Fellow Patients or Those in Similar Needs to Participate in the HKGP.	15 (4.6)	4 (1.2)	48 (14.8)	83 (25.5)	175 (53.8)	79.4 [74.7, 83.4]
The Information Provided During Informed Consent is Helpful.	2 (0.6)	3 (0.9)	41 (14.8)	104 (32.0)	175 (53.8)	85.8 [81.6, 89.2]
I Have More Confidence in Joining the HKGP Knowing that I Can Withdraw at Any time Without Providing a Reason.	4 (1.2)	6 (1.8)	28 (8.6)	73 (22.5)	214 (65.8)	88.3 [84.4, 91.4]
The Genetic Counselling Session Helped Me to Identify What I Needed to Know to Make Decisions about What Would Happen to Me.	2 (0.6)	9 (2.8)	39 (12.0)	88 (27.1)	187 (57.5)	84.6 [80.3, 88.1]
The Genetic Counselling Session was About the Right Length of Time I Needed.	1 (0.3)	2 (0.6)	45 (13.8)	87 (26.8)	190 (58.5)	85.2 [81.0, 88.7]
The Genetic Counselling Session was Helpful to Me.	2 (0.6)	3 (0.9)	37 (11.4)	107 (32.9)	176 (54.2)	87.1 [83.0, 90.3]
I am Confident that My Personal Health Data Will Be Securely Protected.	0 (0)	3 (0.9)	34 (10.5)	85 (26.2)	203 (62.5)	88.6 [84.7, 91.6]
I Feel that Taking Part in HKGP Could Improve My / My Child’s Medical Treatments.	11 (3.4)	11 (3.4)	64 (19.7)	95 (29.2)	144 (44.3)	73.5 [68.5, 78.0]
I Feel that Taking Part in HKGP Could Benefit Others.	1 (0.3)	1 (0.3)	41 (14.8)	83 (25.5)	199 (61.2)	86.8 [82.7, 90.0]
I Feel that Taking Part in HKGP Could Advance Genomic Research in Hong Kong.	2 (0.6)	4 (1.2)	30 (9.2)	82 (25.2)	207 (63.7)	88.9 [85.0, 91.9]

All indicators had positive Net Promoter Scores (NPS) showing that proportion of promoters outnumbered detractors (Fig. 1). All but one achieved NPS above 30. NPS were observed for confidence in joining HKGP (54) and that their personal data would be well protected (51). The lowest NPS at 18 was for agreeing that WGS improves the medical treatment of participants or their children. Out of 325 survey respondents, only 44% (*n* = 143) were promoters who agree with the statement and 26% (*n* = 86) were detractors who disagree with the statement. Fewest detractors was found for satisfaction with their overall experience in HKGP. Only 10% (*n* = 33) reported that they are not satisfied to any extent with the HKGP.

There were few associations between satisfaction indicators with demographic or socioeconomic characteristics in the logistic regression analyses. Of note, when compared with participants with primary education level, those with secondary and tertiary education were less likely to agree that genetic counselling was helpful (OR: 0.02 [95% CI: 0.001–0.41]; 0.02 [95% CI: 0.001–0.51] respectively) (Table 3). They were also less likely to agree that the counselling was the right length of time (OR: 0.12 [95% CI: 0.015–0.83]; 0.12 [95% CI: 0.13–0.98] respectively) and less likely to agree the genetic counselling session helped with decision-making (OR: 0.08 [95% CI: 0.01–0.62]; 0.08 [95% CI: 0.01–0.76] respectively) (*Appendix Table 1, and 2*). Compared to participants age ≤ 18 or caretakers of adolescent patients, participants between 40 and 59 years old were more likely to agree that genetic counselling helped them to identify what they needed to know to make decisions about themselves (OR: 9.00 [95% CI: 1.15–69.92], more likely to agree that genetic counselling was the right length of time (OR: 7.94 [95% CI: 1.11–60.58], and much more likely to agree that genetic counselling was helpful (OR: 26.97 [95% CI: 2.32-313.23].

**Table 3:** Associations between participants characteristics and whether the genetic counselling session was helpful to them

Genetic counselling session was helpful to them	Odds ratio	95% CI	*p*-value
Age Group of Participants			
19 or below	Ref		
20–39	11.49	1.08–122.53	**0.04**
40–59	26.97	2.32–313.23	**0.01**
60 or above	9.59	0.79–116.02	0.08
Gender			
Female	Ref		
Male	1.32	0.51–3.42	0.56
Marital Status			
Not married	Ref		
Married	0.47	0.14–1.57	0.22
Educational Level			
Primary School or Below	Ref		
Secondary School	0.02	0.001–0.41	**0.01**
Tertiary Education or Above	0.02	0.001–0.51	**0.02**
Type of Housing			
Public Housing	Ref		
Private Permanent Housing	1.11	0.36–3.40	0.85
Household Income Level			
≤$19,999	Ref		
$20,000–$29,999	1.62	0.32–8.08	0.56
$30,000–$39,999	4.03	0.50–32.28	0.19
$40,000–$59,999	2.62	0.39–17.70	0.32
≥$60,000	1.69	0.24–11.71	0.60

### Focus groups

We achieved thematic saturation after five semi-structured focus groups with 21 participants. Group sizes ranged from two to six with the median age of participant at 37 years old (age range: 25–69). Extracted content was grouped into six themes (Table 4*)*. Reasons for HKGP participation varied from obtaining a confirmatory diagnosis for their health condition to wanting to help medical research in Hong Kong. While acquiring the genetic test results was a major reason for enrolment, many expressed their desire to aid scientific advances and shorten the diagnostic odyssey of future patients. For example, “I participate to help you (researchers). I have had my blood taken for more than 10 years because of my condition…providing one more sample for you wouldn’t do me harm.” (W, Group 5).

**Table 4:** Key extracted quotes from focus group interview

Theme	Quotes
Reasons for participating in HKGP	*“I think it could help the scientific community… and especially patients in the future.”* (P, Group 3)
	*“I want to give birth to another child*,* I think a comprehensive test like this (HKGP) would be useful…The results would definitely help me in family planning decisions… A lot of current prenatal tests have limitations in conditions that could be screened”* (S, Group 4)
	*“I participate to help you (researchers). I have had my blood taken for more than 10 years because of my condition…Providing one more sample for you wouldn’t do me harm.”* (W, Group 5)
	*“I want to know if my family has the same defective gene…I also want to inform the medical community of my situation and treatment options…But I don’t think there was much personal takeaway involved”* (K, Group 2)
	*“I want to how would my child’s gene influence his development as he grow up…and whether he will pass on the genetic risks to his own children”* (H, Group 2)
Shortening diagnostic odyssey for patients	*“The turnaround time was too long*,* I don’t think the results would be useful to guiding my daughter’s treatment options.”* (P, Group 3)
	*“We are already aware of the diagnosis when our second child was born*,* but it would be nice to have a genetic test result just for our reference… and how it may mutate in the future”* (R, Group 4)
	*“My daughter was diagnosed with type II diabetes more than 20 years ago…but one to two years ago*,* she was informed that the diagnosis should be type I diabetes…We want to know why and what happened”* (V, Group 5)
Comments on overall experience in HKGP	*“The genetic counsellors used pedigree chart to explain results (dominant and recessive traits). It was not too difficult to understand for me and my daughter…but I don’t think it needs to be that in-depth…I just want to get the diagnostic results as swiftly as possible”* (P, Group 3)
	*“I think the pedigree chart from the genetic counsellor was…too complicated. I don’t remember…how my husband and I’s gene…result in my daughter’s condition.”* (V, Group 5)
	*“The blood collection process was quick… I think we need to wait for an hour for other conventional blood draws…but I don’t have to wait for the long queue.”* (B, Group 1)
	*“I have two children…the younger one was recruited into the project. I asked the genetic counsellors if it was possible to enrol my elder child to the project…he could have carried the recessive gene as well*,* right?”* (E, Group 1)
	*“I think the results should be posted on eHealth…When I visit private hospitals*,* they may want to know what were the previous tests conducted in public hospitals”* (S, Group 4)
	*“The medical professionals should share more details about the project…we can help with spreading the information to others…but for now apart from knowing more about genetics of disease*,* I don’t know much about other aspects of the project”* (F, Group 1)
	*“I was filling in my personal information form in a crowded waiting area…There was insufficient space…Some questions asked were rather private*,* I wish I could fill it in a more spacious area so my privacy is protected.”* (V, Group 5)
Reasons not to conduct genetic tests earlier in other settings	*“I think it could be quite costly… I do not think that anyone would go check their gene to understand their risk of disease when their condition is not severe…… I just wanted to know whether my child is a carrier for the disease and be a part of your research project*,* but I would not want to understand more about my genome had it not been this project.” (E*,* Group 1)*
	*“It’s mainly about the cost…also I don’t think there was any medications or procedures that could help with my child’s conditions… We rely on public services…and will comply with tests arranged by medical professionals”* (S, Group 4)
Conflicting experiences	*“It was quite a long procedure… It would be better if they (medical professionals) have told me in advance that the whole procedure (enrolment to study*,* genetic counselling*,* blood taking) could take up to two hours”* (H, Group 2) *“The whole process took 10–15 minutes…It was quite swift…I recall ticking a lot of boxes and I finished the (medical history) form quickly”* (W, Group 5)
	*“I wasn’t happy with the blood taking procedure…That day (enrolment to study) I saw two other parents who had to take leave to provide their blood sample…They were visibly impatient waiting for such a long time.”* (P, Group 3) *“The blood collection process was quick… I think we need to wait for an hour for other conventional blood draws…but I don’t have to wait for the long queue.”* (B, Group 1)
	*“I was in [one of the partnering centres]. The introduction video [on HKGP] was too quick…I had to watch it on my own phone but I couldn’t finish it before see the counsellor… I wish the video could be played on a television.* (B, Group 1) *“The genetic counsellor in [one of the partnering centres] played the video on their tablet computer.”* (C, Group 1)

Despite difficulties in understanding how their genome is analysed or how establishing biobanks could facilitate personalised medicine, participants were generally optimistic that sharing their genome data would allow scientists to better study diseases and devise treatments. Some participants were recruited after receiving a diagnosis or even completing treatment of their underlying conditions; and thus, viewed the HKGP as a complementary test to confirm or support their existing diagnosis, or understand if their condition was genetically related. For example, “We are already aware of the diagnosis when our second child was born, but it would be nice to have a genetic test result just for our reference… and how it may mutate in the future” (R, Group 4).

Participants generally thought that pedigree charts drawn by genetic counsellors, and relevant human phenotype ontology terms used to explain the hereditary nature of diseases were clear and comprehensible. In addition to providing advice and explanation, genetic counsellors were also able to address the emotional needs of participants by offering consolation and reassurance. The genetic counselling session for paediatric cases typically took longer, with extra time needed to explain to parents how the condition manifests as the child grows and the risks to future offspring. Participants stated that previously ambiguous hereditary aspects of their conditions were clarified in the genetic counselling sessions, enhancing literacy on their own health, and increasing understanding of the application of genomic medicine in the healthcare system. For example, “The genetic counsellors used pedigree chart to explain results (dominant and recessive traits). It was not too difficult to understand for me and my daughter…but I don’t think it needs to be that in-depth…I just want to get the diagnostic results as swiftly as possible” (P, Group 3).

Privacy, price, complexity, utility, sensitivity of results, and confidence in test providers were cited as primary reasons not engaging in alternative genetic tests elsewhere. While participants noted that genome sequencing may be common practice overseas for diagnosing rare diseases, some expressed insecurities about the process and unfamiliarity with the organizations involved. Having genome projects conducted by an authoritative body in a healthcare institution provided assurance and enhanced confidence in project engagement. For example, “It’s mainly about the cost…also I don’t think there was any medications or procedures that could help with my child’s conditions. We rely on public services…and will comply with tests arranged by medical professionals” (S, Group 4).

Participants cited reducing the length of time for receiving results as an area of improvement. They also wished for further follow-up with healthcare professionals after the reports and expansion of inclusion criteria to enrol other potential participants/relatives. For example,

“I have two children…the younger one was recruited into the project. I asked the genetic counsellors if it was possible to enrol my elder child to the project…he could have carried the recessive gene as well, right?” (E, Group 1).

Participants also reported heterogeneity in their experiences of clinical procedures and waiting time at partnering centres due to variations in patient journey, care pathways and specialties involved. The duration of genetic counselling and informed consent procedure varied among participants, which might be attributable to differences in participant characteristics and baseline health conditions. “It was quite a long procedure. It would be better if they (medical professionals) have told me in advance that the whole procedure (enrolment to study, genetic counselling, blood taking) could take up to two hours” (H, Group 2); whereas another found “the whole process took 10–15 minutes. It was quite swift. I recall ticking a lot of boxes and I finished the (medical history) form quickly.” (W, Group 5).

## Discussion

We conducted a mixed-methods study to evaluate participant experience in the initial pilot phase of the HKGP, a large-scale population-wide whole genome sequencing project for participants for undiagnosed diseases and hereditary cancer. Most participants were satisfied with their overall experience of the HKGP with almost all indicators achieving high satisfaction rates of 80% or above. The highest support were for satisfaction with overall experience in HKGP and feeling that participation could advance genomic research in Hong Kong. The lowest agreement was feeling that the HKGP could improve their medical treatments. This was consistent with the focus group findings, where many reported that the long turnaround time of diagnostic reports offered limited insights on treatment options. Participants were not offered extra medical consultations regardless of positive diagnosis confirmed by WGS tests. Future interactions could better manage participants’ expectations and highlight other downstream benefits to improve satisfaction.

All indicators had favourable Net Promoter Scores, with excellent outcomes in key areas such as confidence in joining HKGP, advancing research, and data protection. This highlights the project’s potential for positive impact on public perception and trust in genomics, which can be crucial for the successful implementation of genomics in healthcare and research settings [30] by fostering support for data sharing, collaboration, and the responsible use of genomic information [31]. The willingness of participants to recommend the HKGP to those with similar needs indicates the potential for the project to expand its community reach and public impact. As genomic initiatives aim to gather comprehensive and diverse datasets, recommendations from satisfied participants can increase participation rates and include individuals from various backgrounds, thereby enhancing the representativeness and equity of genomic research.

Findings from both the survey and focus group showed the value of genetic counselling sessions in guiding the participant decision-making process. Genetic counselling plays a pivotal role in helping individuals understand the implications of genomic information and make informed choices regarding participation in genomic research [32]. This underscores the importance of genetic counselling in facilitating participant engagement when integrating personalized medicine in routine health services. While levels of satisfaction and perceived utility generally did not significantly differ by participant demographics or socioeconomic characteristics, more highly educated participants with secondary and tertiary education were less likely to agree that genetic counselling was the right length or helpful. Participants with higher educational levels are likely to have prior knowledge about their conditions and better access to other information sources, which potentially reduced the perceived benefits of the genetic counselling. Tailoring the counselling approaches for different ages, education and socioeconomic backgrounds can address the diversity of participant needs and expectations.

Most of the focus group participants stated the primary reason for engaging in the HKGP was to help advance scientific research and benefit future patients rather than direct benefits to themselves or their families. This highlights the altruistic nature of these participants with undiagnosed diseases and hereditary cancer and their willingness to contribute. The findings echo other studies where genome project participants expressed high self-efficacy over engagement in the research study and finding utility even in non-pertinent results [33] with participation were primarily motivated by the desire to help others rather than believing it would help themselves obtain a diagnosis [9]. Nevertheless, participants in non-trial research, including genetic epidemiology, often have expectations of direct benefit, such as being informed of individual results [33–35], necessitating clear communication and education on the potential benefits to each individual. Emphasizing the long-term value of genomic research and its potential to inform personalized treatments may help address this perception gap and increase participant engagement. Promoting the benefits of HKGP participation may be needed for widening enrolment (e.g. inviting participants to share their stories) as a reliance on altruism could be challenging for future roll-out of healthcare services.

The high satisfaction level reported by the participants in the HKGP may be associated with the overall high satisfaction and experience of receiving care in local public hospitals. In 2019, among patients receiving care in local public hospitals setting, 80% rated the care received to be “good” or “excellent/very good” [37] with 78% of survey respondents rated satisfaction of hospital services to be 7/10 or above [38]. Other studies of participant experiences focusing on genetic testing for specific diseases, including Parkinson’s disease [39], hereditary cancers and cardiac conditions [40], have reported high levels of participant satisfaction. Another study found the overall level of decision regret was low among participants in the 100,000 Genomes Project with no significant relationship between receiving a genomic sequencing result and decision regret [41]. The MedSeq Project on WGS in the United States reported that 97% of participants were satisfied with the disclosure of test results from their physician (measured on a composite score of five questions) [42].

Despite the overall high satisfaction level reported by participants in the HKGP, some areas warrant improvement. A common desire among participants was for reducing the reporting time for receiving WGS results. A previous local study highlighted the pre-test counselling should address the expected reporting time in public funded genome sequencing projects, which is especially crucial in prenatal diagnostics as decisions may be time-sensitive for family planning decision [43]. Many research-led genome sequencing projects such as HKGP do not set a specific target turnaround time for test results with the variability in reporting time attributable to the complexity of the cases and the intricate processes involved in genomic analyses. Informed consent processes could be strengthened in future enrolments to address participant expectations on the reporting time of genetic results to ensure participants feel valued and engaged [44]. Some participants valued preventive genetic testing and hoped for an expansion of eligibility criteria in future to allow referral of potential participants/relatives, such as their siblings, nieces/nephews or other distant family members, who might be at risk for undiagnosed conditions. There was also heterogeneity in participant experiences for clinical procedures and waiting time at different stages due to variations in patient journey, care pathways, specialties and partnering centre involved.

Despite the high response rate and the use of mixed-methods evaluation approach, our study had several limitations. The generalizability of focus group findings may be limited to the Chinese population and future studies should include ethnic minority groups and their perspectives. While we included survey responses completed within 30 days of enrolment, participants may still be subjected to recall bias, especially when reporting history of symptom onset that might have occurred several years ago. Although the design of survey instruments and focus group interview guide were reviewed by a panel of key stakeholders, evaluation of additional experience, and insights from patient representatives could improve the survey utility. Analyses assessing validity and reliability of the survey items were not performed as the survey was only intended for this specific project evaluation. The intercoder agreement rate in qualitative analysis was not measured. The online mode of focus group interviews limited the opportunities for inter-participant interactions with a few respondents facing difficulties with audio or video connection. Despite the widespread application of Net Promoter Scores in marketing research and business settings, use in measuring patient experiences is more limited [45] in applicable healthcare settings [24]; thus the Net Promoter Scores are one of a basket of complementary measures of patient experience.

## Conclusions

We conducted a mixed-methods study to evaluate participant experience in the initial phase of the HKGP, a population-wide genome project. Most study participants were highly satisfied with their experience, and nearly all felt that participating in HKGP would benefit others and advance genomic research in Hong Kong. Satisfaction levels were comparable to overseas genomic programmes and locally provided healthcare services. Participants’ major concerns on WGS reporting time could be addressed by strengthening the informed consent process to ensure their expectations align with project implementation. Participants were less certain of direct benefits to themselves. Emphasizing the long-term value of genomic research and its potential for personalized treatments may increase participant engagement.

## Electronic supplementary material

Below is the link to the electronic supplementary material.

Supplementary Material 1

## Data Availability

The data that support the findings of this study are not openly available due to reasons of sensitivity and are available from the corresponding author upon reasonable request.
