# Supplementary material for: Experiences of participants with undiagnosed diseases and hereditary cancers during the initial phase of the Hong Kong genome project: a mixed-methods study
Source: Hum Genomics. 2025 Apr 5;19:36. doi: 10.1186/s40246-025-00746-5 (PMC11972539; doi:10.1186/s40246-025-00746-5)
Supplement: Supplementary file 1 — Supplementary Material 1 [file 40246_2025_746_MOESM1_ESM.docx]

Supplementary Information

Table of Contents

[Appendix 1: Overview of the Hong Kong Genome Project (HKGP)	2](#_Toc193143520)

[Appendix 2. Quantitative Survey	4](#_Toc193143521)

[Appendix 3. Focus Group Interview Guide	10](#_Toc193143522)

[Appendix 4. COREQ checklist	14](#_Toc193143523)

[Appendix Figure 1. HKGP participant workflow	17](#_Toc193143524)

[Appendix Figure 2. Inclusion flowchart	18](#_Toc193143525)

[Appendix Table 1. Associations between whether genetic counselling session helped them to identify what they needed to know to make decisions about what would happen to them	19](#_Toc193143526)

[Appendix Table 2. Associations between participants characteristics and whether the genetic counselling session was about the right length of time they needed	20](#_Toc193143527)

## Appendix 1: Overview of the Hong Kong Genome Project (HKGP)

**About the Project**

HKGP is the first large-scale genome sequencing project in Hong Kong. In collaboration with the Department of Health, the Hospital Authority and local universities, the Project recruits eligible patients and their family members on a voluntary basis and aims to help patients and their families look for the potential disease-causing genetic variants through whole genome sequencing (WGS) analysis.

Launched in 2021, HKGP focuses mainly on diseases and research cohorts that would benefit from WGS. They include undiagnosed diseases, hereditary cancers and cases related to genomics and precision health. Eligible participants must be referred by designated Partnering Centres to join the Project.

Depending on clinical and research needs, data collected from HKGP will be used for clinical applications. Anonymised data will be used for approved medical research under stringent scrutiny, enabling clinicians and scientists to better understand the relationship between genomes and different diseases. The knowledge and experience accumulated will play a crucial role in fostering future medical development in Hong Kong.

Overall coordination and execution of the Project is carried out by:

Health Bureau, The Government of the Hong Kong Special Administrative Region

The Hong Kong Genome Institute

HKGI collaborates with various stakeholders to implement the Project:

The Department of Health, The Government of the Hong Kong Special Administrative Region

The Hospital Authority

Faculty of Medicine, The Chinese University of Hong Kong

LKS Faculty of Medicine, The University of Hong Kong

HKGI has set up Partnering Centres in three hospitals and four referring networks under the Hospital Authority (HA), and is extending collaboration with other HA hospitals, to recruit eligible participants to join the Project:

Partnering Centres:

Hong Kong Children's Hospital

Prince of Wales Hospital

Queen Mary Hospital

Referring Networks:

Alice Ho Miu Ling Nethersole Hospital

Grantham Hospital

Duchess of Kent Children's Hospital at Sandy Bay

The Tung Wah Group of Hospitals

**Project Team**

The Project team comprises clinicians, nurses, scientists, bioinformaticians and genetic counsellors. Their duties include:

Enrolling suitable participants;

Explaining project details and addressing participants’ queries before they sign consent forms;

Collecting samples, performing Whole Genome Sequencing, and analysing the genomic data;

Preparing Whole Genome Sequencing results report for participants; and

Providing genetic counselling and discussing results report with participants

**Aims and objectives**

Most of the rare diseases are either hereditary or due to genetic mutations, and many patients experience early onset in infancy or childhood. Nevertheless, as some genetic disorders do not have distinctive clinical features and are highly variable in presentation, it is often difficult for clinicians to make diagnosis with traditional approaches and routine testing. As a result, patients and their families may end up going through years of diagnostic odyssey. During this prolonged process, patients have to receive numerous tests, make frequent hospital visits and even encounter false leads, creating a huge burden on them and their families. By analysing the sequencing results, especially on rare, hereditary and undiagnosed diseases, clinicians can more easily make precise diagnosis, thereby providing personalised and appropriate treatment to patients and preventive options to their families.

The HKGP aims to recruit 20,000 cases and collect 40,000-50,000 samples for WGS by 2025. Conducting WGS for a large number of patients can help build a database of considerable scale which combines genetic and clinical information. This will not only present immense research value, but also empower clinicians and researchers to uncover the cause of diseases, enabling the development of more effective screening, diagnostic and therapeutic methods.

## Appendix 2. Quantitative Survey

A. Overall satisfaction (3 Questions)

B. Pre-testing genetic counselling (9 Questions)

C. Attitude (5 Questions)

D. Diagnostic odyssey (2 Questions)

E. Demographics (11 Questions)

**A. Overall satisfaction**

Q1 How would you rate your overall experience of the Hong Kong Genome Project (HKGP) thus far?
*Please refer to the HKGP experience (from arriving at the clinic, pre-test counselling, blood sample collection) not the Hospital Authority usual care.*

5 Satisfied (1)

4 Somewhat satisfied (2)

3 Neutral (3)

2 Somewhat dissatisfied (4)

1 Dissatisfied (5)

Q2 Would you recommend fellow patients or those in similar needs to participate in the HKGP?

5 Agree (1)

4 Somewhat agree (2)

3 Neutral (3)

2 Somewhat disagree (4)

1 Disagree (5)

**B. Pre-testing genetic counselling**
(i) Information packages

Q3 I find the following items in the information package helpful:

|  | 5  Agree (1) | 4 Somewhat agree (2) | 3  Neutral (3) | 2 Somewhat disagree (4) | 1  Disagree (5) | N/A (6) |
| --- | --- | --- | --- | --- | --- | --- |
| Information booklet (1) |  |  |  |  |  |  |
| Leaflet (2) |  |  |  |  |  |  |
| Video (3) |  |  |  |  |  |  |
| Other, please specify: (4) |  |  |  |  |  |  |

**(ii) Consent**

Q4 I find the information provided during informed consent is helpful.

5 Agree (1)

4 Somewhat agree (2)

3 Neutral (3)

2 Somewhat disagree (4)

1 Disagree (5)

Q5 I have more confidence in joining the HKGP knowing that I can withdraw at anytime without providing a reason.

5 Agree (1)

4 Somewhat agree (2)

3 Neutral (3)

2 Somewhat disagree (4)

1 Disagree (5)

**(iii) Counselling session**

Q6 The genetic counselling session helped me to identify what I needed to know to make decisions about what would happen to me.

5 Agree (1)

4 Somewhat agree (2)

3 Neutral (3)

2 Somewhat disagree (4)

1 Disagree (5)

Q7 The genetic counselling session was about the right length of time I needed.

5 Agree (1)

4 Somewhat agree (2)

3 Neutral (3)

2 Somewhat disagree (4)

1 Disagree (5)

Q8 The genetic counselling session was helpful to me.

5 Agree (1)

4 Somewhat agree (2)

3 Neutral (3)

2 Somewhat disagree (4)

1 Disagree (5)

**C. Attitude**

Q9 I am confident that my personal health data will be securely protected.

5 Agree (1)

4 Somewhat agree (2)

3 Neutral (3)

2 Somewhat disagree (4)

1 Disagree (5)

Q10 I feel that taking part in HKGP could improve my / my child’s medical treatments.

5 Agree (1)

4 Somewhat agree (2)

3 Neutral (3)

2 Somewhat disagree (4)

1 Disagree (5)

Q11 I feel that taking part in HKGP could benefit others.

5 Agree (1)

4 Somewhat agree (2)

3 Neutral (3)

2 Somewhat disagree (4)

1 Disagree (5)

Q12 I feel that taking part in HKGP could advance genomic research in Hong Kong.

5 Agree (1)

4 Somewhat agree (2)

3 Neutral (3)

2 Somewhat disagree (4)

1 Disagree (5)

**D. Diagnostic odyssey**

Q13 When did you first visit / consult any health-care provider for a suspected genetic disorder?

Within a year (1)

More than a year ago (2)

More than three years ago (3)

More than five years ago (4)

More than ten years ago (5)

**E. Demographics**

Q14 Age of child / patient

<1 (1)

1 - 4 (2)

5 - 9 (3)

10 - 14 (4)

15 - 19 (5)

20 - 24 (6)

25 - 29 (7)

30 - 34 (8)

35 - 39 (9)

40 - 44 (10)

45 - 49 (11)

50 - 54 (12)

55 - 59 (13)

60 - 64 (14)

65 - 69 (15)

70 - 74 (16)

75 - 79 (17)

80 - 84 (18)

>=85 (19)

Q15 Gender

Male (1)

Female (2)

Other (3)

Refused (4)

Q16 Marital status

Married (1)

Divorced (2)

Widowed (3)

Never married (4)

Separated (5)

Refused (6)

Q17 Race / Ethnicity

Chinese (1)

Filipino (2)

Indonesian (3)

Indian (4)

Nepalese (5)

Pakistani (6)

Thai (7)

Japanese (8)

White (9)

Other, please specify: (10) __________________________________________________

Q18 Education level

No schooling / Pre-primary (1)

Primary education (2)

Secondary: Lower secondary (F.1 - F.3) (3)

Secondary: Upper secondary (F.4 - F.4 / DSE / Yi Jin / vocational training included) (4)

Post-secondary: Diploma/Certificate (5)

Post-secondary: Sub-degree course (6)

Post-secondary: Degree course (Degree / Postgraduate or above) (7)

Refused (8)

Q19 Housing type

Public rental housing (1)

Subsidised home ownership housing (2)

Private permanent housing (3)

Non-domestic housing (4)

Temporary housing (5)

Refused (6)

Q20 Monthly household income (in range)

< 2,000 (1)

2,000 - 3,999 (2)

4,000 - 5,999 (3)

6,000 - 7,999 (4)

8,000 - 9,999 (5)

10,000 - 14,999 (6)

15,000 - 19,999 (7)

20,000 - 24,999 (8)

25,000 - 29,999 (9)

30,000 - 39,999 (10)

40,000 - 59,999 (11)

60,000 - 79,999 (12)

80,000 - 99,999 (13)

≥ 100,000 (14)

N/A (15)

Refused (16)

Q21 Do you have any other thoughts / comments? (optional)

________________________________________________________________

Voucher

Do you wish to receive a HK$50 gift coupon?

Yes

No

## Appendix 3. Focus Group Interview Guide

**Focus Group Interview with patients / caretakers of patients**

**Semi-structured Interview Topic Guide**

Aims

To explore understanding of Hong Kong Genome Project (HKGP) (including recall and expectations)

To explore attitudes towards HKGP (including values, benefits and concerns) and motivations for participating HKGP

To explore thoughts, feelings and decision-making about acknowledging the primary results

To investigate information needs regarding participating in HKGP

To understand level of satisfaction with procedures involved in HKGP

To collect reflections on how HKGP could be improved

**Introduction**

Hi. Today is _____________. My name is ______________ and I’m from HKU School of Public Health. I am a Research Assistant from Dr Quan’s team. We are here today to learn about your thoughts and feelings about your participation in the Hong Kong Genome Project.

As you know, we are planning to voice record this interview session so I can listen instead of taking notes, and so that we make sure we understand everything you say correctly. Rest assured that all information collected from this focus group interview would be kept absolutely confidential. All information collected would be discarded within three years from completion of the study.

I am going to be asking you a number of questions about your participation in HKGP, your thoughts and feelings about it, what your expectations were, if there was anything that could have been done differently or done better in the future. Does that sound OK?

Before we start, I would like to confirm that you have read the information sheet about the study that we sent you. Do you have any questions about the information sheet you were sent or anything else relating to this study? If not, we can proceed to embark on the discussion.

[Start recording]

**Diagnostic odyssey**

*(Note: Diagnostic odyssey = time between when a symptom or feature of a genetic or rare disease is noted to the time when a final diagnosis is made.)*

Do you have a definitive diagnosis for your current issue?

🡺 If Yes 🡪

How many years had it been since your first visit / consultation to any health-care provider on your issue.

How many different clinical departments did it take for you to get the diagnosis?

**Satisfaction**

When did you first hear about “HKGP”? How did you hear about it and how much did you know about it?

Prompt: Through the media? HCP? advertisements/ promotions?

Prompt: A project that collects and analyze the genome/ genetic sequences of general population in Hong Kong?

How would you comment on your overall experience in HKGP?

Which part was most satisfactory to you?

Which part did you think could be further refined?

Do you remember why were you invited to participate in HKGP?

Why did you join the HKGP?

Prompt: Recommendations from specialist / primary care [family doctor]/ family members?

Did you have any expectations of joining HKGP/conducting WGS?

Prompt: Primary results (related to your current health issues)

Prompt: Additional findings (disease, carrier, risk to descendants)

How would you describe your overall experience at the hospital?

Prompt: Staff, operation flow, environment etc.

How would you describe your overall experience with the staff?

Are there any potential factors that could facilitate or be barriers to participate in the project? Are there any reasons that prevented you from conducting genetic testing earlier?

Would you recommend others to participate in HKGP? Why or why not?

Do you have any suggestions concerning the operation flow (from recruitment to blood sample collection) of HKGP as you experienced?

**Pre-testing Genetic Counselling**

Concerning the info-pack given to you, what do you think was most relevant/useful to you?

Do you think the consent procedure was clear and explicit?

Do you feel that the HKGP was clearly explained during the informed consent process?

Did you feel you had enough time to think about your participation in the HKGP?

Any additional information that you think should be covered/ included for the informed consent form/ process?

How do you feel about the genetic counselling session (where the genetic counsellor explain what is how genetic conditions might affect you or your family)?

Was the duration sufficient?

Any parts you feel could be better explained? Any parts that you feel were unnecessary?

Did the counselling session help you to make decisions regarding your treatment options/ future?

Is there anything you wish you asked the genetic counsellor?

**(Knowledge +) Attitudes**

What comes to your mind when you hear the phrase genetic testing? To what extent would you say understand the HKGP?

Have you had any genetic testing before participating in HKGP? Have you thought of getting one? Why or why not?

What do you think are the benefits of joining HKGP?

To your health issues/ your treatment options?

To your next of kin?

To other patients?

To scientific community?

To local researchers?

Do you have any concerns? If so, what are your concerns?

How do you currently feel about your decision to have whole genome sequencing done?

**Additional comments**

It seems we have reached the end of discussion today. Do you have any more questions or concerns?

Any other comments, feedback regarding protocol, communication, consent, counselling, impacts, any other aspect of study

Thank you for your kind participation today. If you have any further questions, you can call me at [Research Assistant’s phone number].

## Appendix 4. COREQ checklist

**COREQ (COnsolidated criteria for REporting Qualitative research) Checklist**

A checklist of items that should be included in reports of qualitative research. You must report the page number in your manuscript where you consider each of the items listed in this checklist. If you have not included this information, either revise your manuscript accordingly before submitting or note N/A.

| **Topic** | **Item No.** | **Guide Questions/Description** | **Reported on**  **Page No.** |
| --- | --- | --- | --- |
| **Domain 1: Research team**  **and reflexivity** | | | |
| *Personal characteristics* | | | |
| Interviewer/facilitator | 1 | Which author/s conducted the interview or focus group? | 10 |
| Credentials | 2 | What were the researcher’s credentials? E.g. PhD, MD | N/A |
| Occupation | 3 | What was their occupation at the time of the study? | N/A |
| Gender | 4 | Was the researcher male or female? | 11 |
| Experience and training | 5 | What experience or training did the researcher have? | 11 |
| *Relationship with*  *participants* | | | |
| Relationship established | 6 | Was a relationship established prior to study commencement? | N/A |
| Participant knowledge of  the interviewer | 7 | What did the participants know about the researcher? e.g. personal  goals, reasons for doing the research |  |
|  |  |  | N/A |
|  |  |  |  |
| Interviewer characteristics | 8 | What characteristics were reported about the inter viewer/facilitator?  e.g. Bias, assumptions, reasons and interests in the research topic |  |
|  |  |  | N/A |
|  |  |  |  |

| **Domain 2: Study design** | | | |
| --- | --- | --- | --- |
| *Theoretical framework* | | | |
| Methodological orientation and Theory | 9 | What methodological orientation was stated to underpin the study? e.g. grounded theory, discourse analysis, ethnography, phenomenology, content analysis |  |
|  |  |  | 10 |
|  |  |  |  |
| *Participant selection* | | | |
| Sampling | 10 | How were participants selected? e.g. purposive, convenience,  consecutive, snowball |  |
|  |  |  | 10 |
|  |  |  |  |
| Method of approach | 11 | How were participants approached? e.g. face-to-face, telephone, mail, email |  |
|  |  |  | 10 |
|  |  |  |  |
| Sample size | 12 | How many participants were in the study? | 14 |
| Non-participation | 13 | How many people refused to participate or dropped out? Reasons? | 10 |
| *Setting* | | | |
| Setting of data collection | 14 | Where was the data collected? e.g. home, clinic, workplace | 10 |
| Presence of non-  participants | 15 | Was anyone else present besides the participants and researchers? |  |
|  |  |  | 11 |
|  |  |  |  |
| Description of sample | 16 | What are the important characteristics of the sample? e.g. demographic  data, date |  |
|  |  |  | 14 |
|  |  |  |  |
| *Data collection* | | | |
| Interview guide | 17 | Were questions, prompts, guides provided by the authors? Was it pilot tested? | 10 |
|  |  |  |  |
| Repeat interviews | 18 | Were repeat inter views carried out? If yes, how many? | 10, 14 |
| Audio/visual recording | 19 | Did the research use audio or visual recording to collect the data? | 11 |
| Field notes | 20 | Were field notes made during and/or after the inter view or focus group? | 11 |
| Duration | 21 | What was the duration of the inter views or focus group? | 11 |
| Data saturation | 22 | Was data saturation discussed? | 14 |
| Transcripts returned | 23 | Were transcripts returned to participants for comment and/or correction? | 11 |

| **Domain 3: analysis and findings** | | | |
| --- | --- | --- | --- |
| *Data analysis* | | | |
| Number of data coders | 24 | How many data coders coded the data? | 11 |
| Description of the coding  tree | 25 | Did authors provide a description of the coding tree? |  |
|  |  |  | N/A |
|  |  |  |  |
| Derivation of themes | 26 | Were themes identified in advance or derived from the data? | 11 |
| Software | 27 | What software, if applicable, was used to manage the data? | 11 |
| Participant checking | 28 | Did participants provide feedback on the findings? | 11 |
| *Reporting* | | | |
| Quotations presented | 29 | Were participant quotations presented to illustrate the themes/findings?  Was each quotation identified? e.g. participant number |  |
|  |  |  | 14-16 |
|  |  |  |  |
| Data and findings consistent | 30 | Was there consistency between the data presented and the findings? | 14-16 |
| Clarity of major themes | 31 | Were major themes clearly presented in the findings? | 14-16 |
| Clarity of minor themes | 32 | Is there a description of diverse cases or discussion of minor themes? | 19-20 |

Developed from: Tong A, Sainsbury P, Craig J. Consolidated criteria for reporting qualitative research (COREQ): a 32-item checklist for interviews and focus groups. *International Journal for Quality in Health Care*. 2007. Volume 19, Number 6: pp. 349 – 357

## Appendix Figure 1. HKGP participant workflow

## Appendix Figure 2. Inclusion flowchart

## Appendix Table 1. Associations between whether genetic counselling session helped them to identify what they needed to know to make decisions about what would happen to them

| Genetic counselling session helped them to identify what they needed to know to make decisions about what would happen to them | Odds ratio | 95% CI | p-value |
| --- | --- | --- | --- |
| Age Group of Participants | | | |
| 19 or below | Ref |  |  |
| 20-39 | 5.91 | 0.79 - 42.99 | 0.074 |
| 40-59 | 10 | 1.31 - 76.68 | **0.023** |
| 60 or above | 6.59 | 0.82 - 55.64 | 0.074 |
| Gender | | | |
| Female | Ref |  |  |
| Male | 0.99 | 0.43 - 2.26 | 0.97 |
| Marital Status | | | |
| Not married | Ref |  |  |
| Married | 0.49 | 0.15 - 1.35 | 0.19 |
| Educational Level | | | |
| Primary School or Below | Ref |  |  |
| Secondary School | 0.10 | 0.01 - 0.75 | **0.03** |
| Tertiary Education or Above | 0.12 | 0.01 - 0.99 | 0.06 |
| Type of Housing | | | |
| Public Housing | Ref |  |  |
| Private Permanent Housing | 0.90 | 0.32 - 2.47 | 0.84 |
| Other Types of Housing | 1.78 | 0.38 - 14 | 0.51 |
| Household Income Level | | | |
| ≤$19,999 | Ref |  |  |
| $20,000–$29,999 | 1.73 | 0.44 - 6.53 | 0.42 |
| $30,000–$39,999 | 5.33 | 0.95 - 42.79 | 0.072 |
| $40,000–$59,999 | 2.39 | 0.51 - 11.45 | 0.27 |
| ≥$60,000 | 1.71 | 0.35 - 8.04 | 0.51 |

## Appendix Table 2. Associations between participants characteristics and whether the genetic counselling session was about the right length of time they needed

| Genetic counselling session was about the right length of time they needed | Odds ratio | 95% CI | p-value |
| --- | --- | --- | --- |
| Age Group of Participants | | | |
| 19 or below | Ref |  |  |
| 20-39 | 4.18 | 0.58 - 30.69 | 0.15 |
| 40-59 | 8.95 | 1.23 - 69.21 | **0.03** |
| 60 or above | 5.02 | 0.65 - 41.83 | 0.12 |
| Gender | | | |
| Female | Ref |  |  |
| Male | 1.51 | 0.68 - 3.46 | 0.32 |
| Marital Status | | | |
| Not married | Ref |  |  |
| Married | 1.23 | 0.47 - 3.04 | 0.66 |
| Educational Level | | | |
| Primary School or Below | Ref |  |  |
| Secondary School | 0.12 | 0.01 - 0.81 | **0.04** |
| Tertiary Education or Above | 0.11 | 0.01 - 0.91 | **0.05** |
| Type of Housing | | | |
| Public Housing | Ref |  |  |
| Private Permanent Housing | 0.82 | 0.32 - 2.08 | 0.68 |
| Other Types of Housing | 5.13 | 0.78 - 108.63 | 0.16 |
| Household Income Level | | | |
| ≤$19,999 | Ref |  |  |
| $20,000–$29,999 | 1.17 | 0.31 - 4.11 | 0.81 |
| $30,000–$39,999 | 2.42 | 0.51 - 12.46 | 0.27 |
| $40,000–$59,999 | 2.14 | 0.46 - 9.85 | 0.32 |
| ≥$60,000 | 3.75 | 0.70 - 20.42 | 0.12 |
